# Respectful maternity care in Ethiopian public health facilities

**DOI:** 10.1186/s12978-017-0323-4

**Published:** 2017-05-16

**Authors:** Ephrem D. Sheferaw, Eva Bazant, Hannah Gibson, Hone B. Fenta, Firew Ayalew, Tsigereda B. Belay, Maria M. Worku, Aelaf E. Kebebu, Sintayehu A. Woldie, Young-Mi Kim, T. van den Akker, Jelle Stekelenburg

**Affiliations:** 1 Jhpiego, Addis Ababa, Ethiopia; 2 Jhpiego, Baltimore, USA; 3 Ethiopian Midwives Association, Addis Ababa, Ethiopia; 4 Ministry of Health, Addis Ababa, Ethiopia; 5 Department of Obstetrics, Leiden University Medical Center, Leiden, The Netherlands; 6 Leeuwarden Medical Centre, Leeuwarden, The Netherlands; 7 University Medical Centre Groningen, University of Groningen, Groningen, The Netherlands

**Keywords:** Respectful maternity care, Mistreatment of women, Labor and delivery, Birth companion, Birth positioning, Ethiopia, Health facility

## Abstract

**Background:**

Disrespect and abuse of women during institutional childbirth services is one of the deterrents to utilization of maternity care services in Ethiopia and other low- and middle-income countries. This paper describes the prevalence of respectful maternity care (RMC) and mistreatment of women in hospitals and health centers, and identifies factors associated with occurrence of RMC and mistreatment of women during institutional labor and childbirth services.

**Methods:**

This study had a cross sectional study design. Trained external observers assessed care provided to 240 women in 28 health centers and hospitals during labor and childbirth using structured observation checklists. The outcome variable, *providers’ RMC performance*, was measured by nine behavioral descriptors. The outcome, *any* mistreatment, was measured by four items related to mistreatment of women: physical abuse, verbal abuse, absence of privacy during examination and abandonment.

We present percentages of the nine RMC indicators, mean score of providers’ RMC performance and the adjusted multilevel model regression coefficients to determine the association with a quality improvement program and other facility and provider characteristics.

**Results:**

Women on average received 5.9 (66%) of the nine recommended RMC practices. Health centers demonstrated higher RMC performance than hospitals. At least one form of mistreatment of women was committed in 36% of the observations (38% in health centers and 32% in hospitals).

Higher likelihood of performing high level of RMC was found among male vs. female providers ($\widehat{\beta}=0.65$, *p* = 0.012), midwives vs. other cadres ($\widehat{\beta} = 0.88$, *p* = 0.002), facilities implementing a quality improvement approach, Standards-based Management and Recognition (SBM-R^©^) ($\widehat{\beta}=1.31$, *p* = 0.003), and among laboring women accompanied by a companion $\widehat{\beta} = 0.99$, *p* = 0.003). No factor was associated with observed mistreatment of women.

**Conclusion:**

Quality improvement using SBM-R^©^ and having a companion during labor and delivery were associated with RMC. Policy makers need to consider the role of quality improvement approaches and accommodating companions in promoting RMC. More research is needed to identify the reason for superior RMC performance of male providers over female providers and midwives compared to other professional cadre, as are longitudinal studies of quality improvement on RMC and mistreatment of women during labor and childbirth services in public health facilities.

## Plain English summary

Disrespect and abuse of women during institutional childbirth services is one of the deterrents to utilization of maternity care services in Ethiopia and other low- and middle-income countries. This paper describes the level of respectful maternity care (RMC) and mistreatment of women reported by women who gave childbirth in health facilities in Ethiopia, and identifies associated factors.

Trained external observers assessed care provided to 240 women in 28 health centers and hospitals during labor and childbirth using structured observation checklists. The outcome variable, *providers’ RMC performance*, was measured by nine behavioral descriptors. The outcome, *any* mistreatment of women, was measured by four items indicative of mistreatment of women: physical abuse, verbal abuse, absence of privacy during examination and abandonment.

Women on average received six of the nine recommended RMC practices. Health centers demonstrated higher RMC performance than hospitals. Any form of mistreatment of women was committed in more than two-thirds of the observations. Higher likelihood of performing high level of RMC was found among male providers vs. female, midwives vs. other cadres, facilities implementing a quality improvement approach, Standards-based Management and Recognition (SBM-R^©^and among laboring women accompanied by a companion. No factor was associated with observed mistreatment of women during institutional labor and childbirth services. Quality improvement using SBM-R^©^ and having a companion during labor and delivery were associated with RMC. Policy makers need to consider the role of quality improvement approaches and accommodating companions in promoting RMC. More research is needed to identify the reason for superior RMC performance of male providers over female providers and midwives compared to other professional cadre.

## Background

Following the growing evidence on women’s experience of mistreatment of women during pregnancy and childbirth across the globe, the World Health Organization (WHO) released a statement on prevention and elimination of disrespect and abuse (D&A) during facility-based childbirth [1]. The statement advocates for governments and development partners to initiate, support and sustain programs designed to address quality of Maternal and Newborn Health (MNH) services with a strong emphasis on the provision of respectful maternity care (RMC) as an essential component of quality of care [1]. The White Ribbon Alliance defines RMC as an approach that emphasizes the positive interpersonal interactions of women with health care providers and staff during labor, delivery, and the postpartum period. Absence of D&A by health care providers and other staff alone is not sufficient for provision of RMC; the RMC definition calls for fostering positive staff attitudes and behaviors that are conducive to improved satisfaction of women with their birth experience [2]. Assessing the status of mistreatment of women in health facilities will inform programs engaged in promotion of RMC without losing sight in reducing mistreatment of women.

In Ethiopia, the proportion of childbirths attended by a Skilled Birth Attendant (SBA) in 2014 was 15%, compared to 50–53% in other Sub-Saharan African countries, especially in East Africa [3, 4]. In many countries, one of the reasons for low rate of childbirth assisted by SBA is absence of RMC and the actual and perceived high D&A committed by health providers [5–8]. As elsewhere, in Ethiopia, D&A is a deterrent to women seeking childbirth in health facilities. A 2014 synthesis of evidence from 65 studies on the barriers of facility-based delivery in low-and middle-income countries showed many individual, community, and health system related factors, including mistreatment of women, geographic accessibility, health care costs, perceptions of quality, cultural and personal preferences, and education, contributed to low SBA rates [8]. This synthesis also noted that health professionals working at health facilities were not sensitive to women’s privacy and showed little care in giving them psychological support when women requested it [8, 9]. A 2014 study conducted in Addis Ababa at two health centers and one university teaching hospital found that 78% of women reported having experienced some form of D&A [10]. There was also discrepancy between hospitals and health centers.

The Ethiopian Ministry of Health is highly committed to increasing the rate of SBA-assisted deliveries in health facilities; their health sector transformation plan (HSTP) has a target of 90% skilled birth attendance rate and a reduction of the maternal mortality ratio (MMR) from 420/100,000 live births in 2015 to 199/100,000 live births by 2020 [11]. The focus in the Health Sector Development Plans III and IV (implemented during 2005–2014) to achieve a higher rate of attended births at health facilities and a reduced MMR was mainly focused on bringing services closer to the community. Ethiopia’s Ministry of Health acknowledges, however, that provision of RMC is also a key intervention to bring unreached women to health facilities for maternity care services and thus, an important component in achieving their 2020 goals. To date, some efforts have been made to integrate RMC in the in-service training packages for MNH care, particularly Basic Emergency Obstetrics and Newborn Care (BEmONC) training. The BEmONC training package encourages providers to deliver services that are acceptable to women, that empower women and their families to become active participants in care, protect the rights of women, ensure that all healthcare staff use positive interpersonal communication with women and companions and promote provision of emotional, psychological, and social support to women [12].

This analysis draws on data from a larger study designed to assess the Standards-Based Management and Recognition (SBM-R^©^) quality improvement approach that was implemented for two years in Ethiopia. SBM-R^©^is a quality improvement approach developed by Jhpiego that sets evidence-based performance standards and then empowers health-care managers and providers to assess and address gaps between actual and desired performance at their facility [13]. The SBM-R^©^ approach to quality improvement comprises four steps:1) defining evidence-based and locally relevant standards 2) assessing the gap between desired and actual performance, designing and implementing interventions to close this gap within health facilities3) periodically measuring progress towards desired performance and 4) rewarding performance [14–17].

The objectives of this manuscript are a) to measure the prevalence of RMC and mistreatment of women in hospitals and health centers and b) to identify factors associated with the observed RMC and mistreatment of women in Ethiopia, including facility- and provider-related factors.

## Methods

### Study design

This study used data form the SBM-R^©^ quality improvement approach evaluation. This analysis used cross-sectional data combining both SBM-R^©^ intervention and matched comparison sites. This manuscript focused on the observation of care data and in particular, the respectful maternity care elements.

## Study setting

Ethiopia uses a three-tier health structure of primary, secondary and tertiary levels. The primary level includes health centers with their satellite health post and primary hospitals. In the secondary and tertiary level, general hospitals and specialized hospitals are included [11].

Maternal and Child Health Integrated Program (MCHIP) implemented by Jhpiego used SBM-R^©^ as part of a comprehensive package of interventions aimed at improving quality of maternal and newborn health including RMC in Ethiopia for two years between 2002 and 2003. The study was conducted in the four regions of the country namely, Tigray, Amhara, Oromia and SNNP regions. A total of 28 urban and peri-urban health facilities six referral hospitals and 22 health centers were selected.

Half of the facilities participated in the study (three hospitals and eleven health centers) had implemented SBM-R^©^ approach.

### Sample size

The unit of analysis for this study was each observation, which represents a unique woman. Providers may have cared for multiple women during the observation period. Sample size for labor and delivery observation in the larger SBM-R^©^ evaluation study was calculated to detect a minimum of 20% difference in performance of Active Management of Third Stage of Labor (AMSTL) between SBM-R^©^intervention and comparison facilities, with 80% statistical power, 95% level of confidence and the recommended value of 1% intraclass correlation coefficient for median value of primary health care research [18]. The performance of AMSTL for comparison sites was set as 29% using a previous MCHIP quality of care study [19]. The final sample size was 240 women. A total of 117 providers who were on duty during data collection period were invited for observation. All women who came for labor and delivery and postnatal care were invited for observation.

## Data collection

The study used a structured observation of the provider-client interaction during normal labor and delivery services. Trained assessors were clinicians (bachelor and master’s degree level midwives and health officers) and national level BEmONC trainers who were external to the facility, recruited from regions other than their own. Each assessor went through a one-week study training workshop. Data were collected in July and August, 2014. Assessors observed midwives, nurses and health officers who were providing labor and delivery services during day and night. The assessors were not intervening with the care provided to women. In an event where the assessor deemed the safety or life of the mother or newborn in danger, or where the client’s status was deteriorating, the assessors were trained to alert a senior clinician to intervene. The observation of women started in the second stage of labor and continued to two hours post-delivery. Two assessors were assigned per facility and each covered two eight hour shifts per day. In each health facility between two and 11 women were observed within two to five days. In 16 of the facilities assessed, 11 women were observed; in the remaining 12 health centers, between two and nine women were observed. The median number of women observed per facility was 11.

## Data quality

To ensure data quality, the study coordinator oversaw the data collection process, closely communicating with the principal investigator and supervisors. Each day, supervisors checked the completeness of observational data collected.

## Measures

The two outcomes of interest (dependent variables) were ‘any mistreatment of women’ and total number of RMC descriptors practiced by providers. Each element comprising these outcome measures was recorded as dichotomous (observed or not observed). The providers’ mistreatment of women and RMC structured observation checklist was adapted from the MCHIP quality of care checklist. The larger study was validated in five countries, including Ethiopia [20].

The structured RMC observation checklist included 9 items that described desirable provider behaviors. The desirable provider behaviors included: (1) receiving and greeting the pregnant women, (2) explaining each step of the examination, (3) encouraging women to ask questions, (4) responding to women and their companions politely when they asked questions, (5) explaining to women what will happen in labor, (6) encouraging women to walk and change position, (7) ensuring light eating, (8) asking women which position they would like to deliver in and (9) allowing women to give birth in the position they want. The outcome variable was the sum of the nine equally weighted RMC behaviors practiced for each observation and ranged from 0 to 9.

The undesirable provider behaviors reflecting mistreatment of women included 4 items: (1) physical abuse (slapping or hitting women during labor), (2) verbal abuse (making insults or threatening women and or their companions), (3) the absence of privacy during examination and (4) abandonment (leaving women alone during labor). In the Bohren et, al. (2015) typology of mistreatment of women during childbirth, the four items are mapped with four of the seven third-ordered themes [21]. The outcome variable, ‘any mistreatment of women’ was dichotomous requiring a ‘yes’ or ‘no’ response. ‘Yes’ was marked if any of the above behaviors was observed. RMC ranged between 0 and 100%.

## Data management and analysis

Cleaned observation data were entered twice into CS Pro 5.0 [22]. Data discrepancies were resolved and the data were exported to STATA 13.0 for further analysis [23].

Chi square test for categorical variables were used to compare health workers’ practice of mistreatment of women with facility types (health centers and hospitals). Independent samples *t*-test were used to compare health workers’ RMC practices with facility types. Socio-demographic characteristics of observed health workers and facility characteristics were reported using frequency and percentage disaggregated by facility type. Tests of proportions and relationships between mistreatment of women, RMC and socio-demographic variables were computed at 5% level of significance.

Multivariable, multilevel linear regression for the continuous outcome variable, total RMC score, and multivariable, multilevel logistic regression analysis for the categorical outcome, any mistreatment of women, were used because observation data are hierarchical (i.e. clients are nested within providers, providers are nested with in health facilities). Also, the use of flat (non-clustered) models could underestimate the standard errors of the effect sizes, which consequently can affect decision on null hypothesis. In such data, women observed within same health facility may be more similar to each other than women observed in other health facilities.

Three steps were used to fit multilevel logistic regression and multilevel linear regression models. First, the null, unadjusted model (without predictors) helped determine whether multilevel modeling was needed. Second, bivariate logistic and linear regression models were fitted to identify potential predictors of occurrence of mistreatment of women and practice of RMC for multivariable analysis. Third, multivariable logistic and linear regression models were fitted to identify predictors of occurrence of mistreatment of women and practice of RMC. The interclass correlation coefficients (ICC) for the null model and multivariable model were calculated and used to evaluate the variations explained by facility and provider cluster effects on the outcome variables [24]. For selection of candidate variables for the multivariate model, *p*-value of less than 0.25 was used.

The fixed effect sizes of individual and facility-level factors on the total RMC scores were expressed using regression coefficient (β), adjusted regression coefficients ($\widehat{\beta}$), the 95% Confidence Interval (CI) and *p*-values. Whereas, the fixed effect sizes of individual and facility-level factors on the observed practice of mistreatment of women were expressed using the crude odds ratio (COR), adjusted odds ratio (AOR), the 95% Confidence Interval (CI) and *p*-values.

## Ethics

The study protocol was reviewed and approved by the National Ethics Review Committee (NERC) at the Ministry of Science and Technology in Ethiopia. The Johns Hopkins Bloomberg School of Public Health Institutional Review Board in Baltimore, Maryland, USA, indicated the study was exempt from oversight under U.S. legislation, 45 CFR 46.101(b). Recruitment of women and consent process were conducted immediately after arrival at the facility. In this study, each woman interviewed, observed and each provider observed gave informed written consent prior to participation.

## Results

We observed 240 women (175 in health center and 65 in hospitals) during labor and childbirth. The observed deliveries were managed by 117 providers in 28 facilities. An average of two women were observed per provider (range of one to eight). The median number of women observed per facility was 11.

Females provided care in three-fourths of the observations (73% or *n* = 174). Most observations were of deliveries with midwives (78%, *n* = 187), and midwife-assisted deliveries were observed more in hospitals than health centers (94% vs. 72%, *p* < 0.001). Health workers allowed a support person during labor in 84% of observations (86% in health centers and 81% in hospitals) (Table 1).

**Table 1:** Characteristics of Labor and Delivery Observations, by Facility Type (Observations as the unit of analysis)

	Total Observations		Health Center observations		Hospital observations		*p*-value (Chi-Square)
	%	N	%	N	%	N	
Provider characteristics							
Sex							
Male	*27*	65	*32*	55	*15*	10	0.009*
Female	*73*	174	*68*	119	*85*	55	
Profession							
Midwife	*78*	187	*72*	126	*94*	61	<0.001*
Others (Nurse, doctor, health officers)	*22*	53	*28*	49	*6*	4	
Region							
Tigray	*18*	44	*13*	22	*34*	22	0.864
Amhara	*27*	65	*37*	65	*0*	65	
Oromiya	*28*	66	*25*	44	*25*	22	
SNNPR	*27*	65	*25*	44	*25*	21	
Support person allowed during labor	*84*	195	*86*	144	*78*	51	0.179

*. P-value significant at 0.05 level

As shown in Table 2, the observations were conducted in 28 health facilities (22 health centers and 6 hospitals). The health centers included for the observation had an average of 646 annual deliveries and the hospitals had an average of 1,974 annual deliveries. On average, health centers had 5.5 beds with a standard error of 0.3 whereas hospitals had 159 beds with a standard error of 4.9. Health centers had an average of 5.8 MNH staff with a standard error of 0.2 and hospitals had an average of 17 MNH staff with a standard error of 0.3.

**Table 2:** Characteristics of Facilities Participated in Labor and Delivery Observations

Facility characteristics	Total (*N* = 28)	Health Centers (*N* = 22)	Hospitals (*N* = 6)	*p*-value (independent sample *t*-test)
	mean (SE)	mean (SE)	mean (SE)	
Annual deliveries	1006 (53)	646 (27)	1974 (97)	0.012*
Number of beds	50 (4.8)	5.5 (0.3)	159 (4.9)	<0.001*
Number of MNH staff	9 (0.4)	5.8 (0.2)	17 (0.3)	<0.001*
Number of BEmONC trained staff	4 (0.3)	2.3 (0.1)	9 (0.7)	0.069

*. P-value significant at 0.05 level

## Prevalence of respectful maternity care

The most frequently practiced RMC element was ensuring that women take light food, occurring in 83% (*n* = 193) observations. The least practiced item was asking women’s preference of birth position, observed in only 29% (*n* = 68) of the observations. Health centers performed better than hospitals in all nine practices and the differences were statistically significant in the following five practices: receiving and greeting women, encouraging women to ask questions, encouraging walking and changing positions, ensuring women have taken light food and allowing women to give birth in the position she prefers. On average 5.9 (66%) of the 9 recommended RMC descriptors were performed; the average performance in health centers was significantly higher compared to health centers 6.2 (69%) and in hospitals 5.3 (59%), *p* = 0.007 (Table 3).

**Table 3:** Prevalence of RMC services during labor and delivery, by Facility Type, Ethiopia 2014 (*N* = 240 observations)

	Total (*n* = 240)		Health Center (*n* = 175)	Hospital (*n* = 65)	*p*-value
Provider:	**%**	No.	**%**	**%**	
…receives and greets the pregnant women	*77*	181	*82*	*63*	0.002*
Don’t know or missing	*2*	5	*3*	*0*	
…explains each step of the examination to the women	*65*	153	*69*	*57*	0.092
Don’t know or missing	*3*	6	*3*	*0*	
…encourages the women to ask questions	*39*	90	*44*	*26*	0.015*
Don’t know or missing	*3*	7	*4*	*0*	
…responds to a women/companion question politely	*72*	167	*74*	*68*	0.328
Don’t know or missing	*4*	9	*5*	*0*	
…explains what will happen in labor to women	*81*	188	*78*	*88*	0.107
Don’t know or missing	*3*	8	*5*	*0*	
…encourages women to walk and change position	*69*	162	*73*	*59*	0.027*
Don’t know or missing	*3*	6	*3*	*0*	
…at least once ensures if she has taken light food	*83*	193	*87*	*73*	0.011*
Don’t know or missing	*3*	8	*3*	*3*	
…asks women which position she would like to deliver	*29*	68	*33*	*20*	0.052
Don’t know or missing	*3*	8	*5*	*0*	
…allowed to give birth in the position she wants	*38*	85	*42*	*27*	0.029*
Don’t know or missing	*6*	15	*8*	*2*	
Average number of RMC Practices performed	*66*	*5.9*	*69*	*59*	0.007*

*. P-value significant at 0.05 level

## Observed practice of mistreatment of women

Of the total 240 observations, in 36% (*n* = 87) at least one form of mistreatment of women was observed (Table 3). The element with the highest prevalence was abandonment or being left alone, 19% (*n* = 43). Verbal abuse occurred in 8% (*n* = 18) of the observations. No statistically significant difference was observed between hospitals and health centers in observed prevalence of these elements of mistreatment of women (Table 4).

**Table 4:** Prevalence of mistreatment of women during labor and delivery, by Facility Type

	Total		Health Center		Hospital		*p*-value
Item	%	No.	%	No.	%	No.	
Physical abuse	*9*	21	*9*	15	*10*	6	*0.973*
Verbal abuse	*8*	18	*6*	10	*12*	8	*0.117*
Privacy violated	*17*	40	*17*	29	*17*	11	*0.951*
Abandonment: or being left alone	*19*	43	*19*	32	*17*	11	*0.745*
*Summary Outcome*							
*Any mistreatment of women*: At least one form of mistreatment of women	*36*	87	*38*	66	*32*	21	*0.436*

Table 5 describes results from multivariate linear regression analysis of facility and provider related factors associated with total RMC score. Midwives were more likely to have higher total RMC score compared to other providers (nurses, health officers and doctors) [$\widehat{\beta} = 0.88$, 95% CI (0.32, 1.44); *p* = 0.002]. The coefficient was higher among male than female providers [$\widehat{\beta} = 0.65$, 95% CI (0.15, 1.16); *p* = 0.012]. Facilities that implemented SBM-R approach had a higher RMC score [$\widehat{\beta}=1.31$, 95% CI (0.434, 2.19), *p* = 0.003]. Women were more likely to have higher RMC scores when birth companions were allowed in labor and delivery rooms [$\widehat{\beta} = 0.99$, 95% CI (0.335, 1.63), *p* = 0.003). Health centers had a higher RMC score compared to hospitals, although this finding was not statistically significant.

**Table 5:** Factors Associated with Provision of RMC in Labor and Delivery in Bivariate and Multivariable Multi-level Regression Models (Observation): Outcome variable: Number of RMC practices performed

Predictor	Bivariate			Multivariate		
	Coefficient ($\beta$)	95% CI	*p*-value	Adjusted Coefficient ($\widehat{\beta}$)	95% CI	*p*-value
Cadre						
Midwife (Ref: Others (Nurses, doctors, health officers)	0.75	0.20,1.30	0.007	0.88	0.32,1.44	0.002*
Provider gender						
Female (Ref: Male)	-0.44	-0.98, 0.09	0.107	-0.65	-1.16, -0.15	0.012*
Facility type						
Health center (Ref: Hospital)	0.94	-0.534,2.15	0.237	0.92	-0.106, 1.95	0.079
QI Intervention status						
Intervention (Ref: Comparison)	1.29	0.25, 2.34	0.016	1.31	0.43, 2.19	0.003*
Companion encouraged						
Yes (Ref: No)	1.03	0.358,1.71	0.003	0.99	0.335, 1.63	0.003*
Region						
Amhara (Ref: Tigray)	0.68	-0.94, 2.31	0.409			
Oromiya	-1.22	-2.9, 0.46	0.155			
SNNPR	-0.8	-2.37, 0.77	0.319			
Annual number of deliveries	-0.0002	-.001,0.0005	0.553			
Number of MNH staff	-0.030	-0.134,0.074	0.57			
Number of BEmONC trained staff	0.026	-0.133, .186	0.747			

Notes. Provision of RMC services during labor and delivery was defined as mean percentage score on a total of 10 practices

Variables included in the multivariate are those with *p*- values of less than 0.25 at bivariate level

*. *P*-value significant at 0.05 level. OR, adjusted coefficient, 95% CI, and confidence interval. *Ref*, reference group

Table 6 shows results from multi-level multivariable logistic regression analysis of any mistreatment of women observed in labor and delivery observations as an outcome and provider’s facility and provider characteristics variables as explanatory variables. None of the hypothesized provider and facility-related characteristics were associated with observed mistreatment of women.

**Table 6:** Factors Associated with Any Mistreatment of Women in Labor and Delivery in Bivariate and Multivariable Multi-level Regression Models (Observation), (*n* = 240): Outcome variable: Any Mistreatment of Women

Predictor	Bivariate			Multivariate		
	COR	95% CI	p-value	AOR	95% CI	*p*-value
Cadre						
Midwife (Ref: Others (Nurses, doctors, health officers)	0.48	0.15,1.57	0.226	0.56	0.13,2.44	0.441
Provider gender						
Female (Ref: Male)	0.85	0.29,2.49	0.769			
Facility type						
Hospital (Ref: Health center)	0.65	0.06,7.22	0.724			
Intervention status						
Comparison (Ref: Intervention)	5.41	0.80,5.41	0.083	4.65	0.51,42.5	0.174
Companion encouraged						
Yes (Ref: No)	0.422	0.11, 1.60	0.205	0.48	0.11,2.06	0.324
Annual number of deliveries	0.99	0.99,1.0	0.126	1.00	0.99,1.0	0.082
Number of beds	0.99	0.97,1.01	0.445			
Number of MNH staff	0.98	0.81,1.20	0.908			
Number of BEmONC trained staff	0.73	0.51,1.03	0.078	1.33	0.71,2.50	0.368
Region						
Amhara (Ref: Tigray)	0.28	0.01,5.7	0.408	0.23	0.01, 5.08	0.350
Oromiya	8.24	0.41,165.8	0.168	7.67	0.38, 156.03	0.185
SNNPR	7.29	0.45,117.9	0.162	10.88	0.62, 192.17	0.103

Notes. Any mistreatment of women during labor and delivery was defined as mean percentage score on a total of 10 aspects. *COR* crude odds ratio, *AOR* adjusted odds ratio, *95% CI* confidence interval, *Ref* reference group

## Discussion

In this study, carried out in hospitals and health centers of four regions of Ethiopia, labors and births were observed. The analysis revealed the prevalence of RMC and mistreatment of women in hospitals and health centers and identified factors associated with the observed RMC and mistreatment of women.

### Respectful maternity care

On average, a woman received two-thirds of the aspects of RMC assessed. We discuss some of the practices that were least likely to be observed in our study and showed significant variation between hospitals and health centers.

#### Allowing women to choose preferred birthing position

Providers’ practice of allowing women to choose their preferred birth positioning occurred at the lowest frequency of all the desired behaviors; only about two in five women in health centers and one in five women in hospitals were given choices for delivery position. Quality statement 6.2 of the WHO standards for improving quality of maternal and newborn care in health facilities states that every woman should receive support to encourage her to adopt the position of her choice during labor [25]. Bohren et al’s [26] systematic review of barriers to institutional delivery found that being asked to adopt unfamiliar birthing positions and having no control over choice of birthing position are important reasons why some women prefer home deliveries. In our study, the practice of allowing preferred positions was significantly higher in health centers than in hospitals. A possible reason for this discrepancy may be the relatively higher client volumes and lower staff-to-patient ratios in hospitals, which may impede providers’ ability to offer more individualized care. The low level of practice of allowing women to choose their preferred birthing position could be attributed to the fact that facilities usually do not have physical structures for alternative birth positions (i.e., suitable delivery couches or floor space for squatting positions). For example, a study in Afar region in Ethiopia showed women preferred a sitting position for delivery but delivery beds that have space for a semi-sitting position were not available [27]. Providers’ lack of training on alternate birth positions, particularly during their pre-service practicum, may also explain why some do not allow women to deliver in their preferred position. Health workers in a study in Bangladesh and Uganda reported that they had not been trained to deliver women in positions other than lying at their backs and thus did not feel confident to do so [28, 29].

#### Light eating

A majority of women were permitted to take light food during labor and delivery, with health centers encouraging this more frequently than hospitals. The practice occurred much more frequently than in a previous study in Ethiopia in 2012 that reported only 40% of women were allowed food or fluid intake during labor and delivery [20]. The reason for the higher rate in our study could be the result of exposure of providers to the in-service BEmONC training that includes an RMC session focused on interpersonal communication skill of providers, respecting culture, belief and values of clients [30].

#### Birth companions

Birth companions can improve experiences of women during labor and delivery; this is articulated in a statement by the World Health Organization [31]. One of the promising findings of this study was health workers’ frequent practice of allowing a support person to be with women during labor. Four in five women were allowed to have a support person during labor, with no significant difference between health centers and hospitals. The finding was promising compared to another qualitative study, in Tanzania, that reported women felt ignored and neglected during child birth because family members or companions were not allowed to provide support [32]. Similarly, a study conducted in Jordan also revealed that women felt dissatisfied with the health system when they were not allowed to have a support person in delivery room [33].

#### Provider and facility factors

Several socio-demographic and health facility factors were found to be related to observed RMC practices. First, the type of health worker was significantly associated with provision of RMC care; midwives were better RMC service providers compared to nurses, health officers and doctors perhaps because their training focuses primarily on maternity care. In Ethiopia MNH service is provided by midwives, nurses, health officers and doctors. A Cochrane review on midwife-led models of care for childbirth in high income countries showed that midwife-led care was beneficial particularly for normalizing and humanizing childbirth [34].

Surprisingly, male providers were observed engaging in RMC practices more frequently than female providers. This finding is difficult to interpret and runs counter to stereotype of women being more empathic and caring than men. A clue from a study of nurses’ abuse of patients in South Africa concluded that female nurses deployed violence against patients in their work as a means of creating social distance and maintaining fantasies of identity and power in their continuous struggle to assert their professional and middle class identity [5]. A literature review on barriers to quality midwifery care discussed the triple burdens faced by female midwives: (1) reproductive (childbearing), (2) productive (economic), and (3) community management (e.g. unpaid work in support of the community). The effect of social, economic and professional barriers resulted in moral distress and burn out, which may have led to abusive behavior [35]. The sex and professional disparity in the provision of RMC calls for strengthened intervention starting from teaching institutions, in-service training and health program administration to institutionalize provision of RMC by all providers male and female. This is also in line with MOH’s health sector transformational agenda of creating a caring, respectful and companionate health professionals [36].

The third factor that affected provision of RMC was the presence of birth companion. Women were more likely to receive RMC when birth companions were allowed in labor. Presence of birth companions helped the women receive emotional and physical support and comfort from their loved ones, and removed some of the burden from health workers. Respondents in studies in Tanzania discussed how birth companions assisted and encouraged women, because providers were absent [32, 37]. The WHO Safe Birth checklist also mentions companions in the context of calling providers for help when needed [38].

The final factor that showed a significant relationship to the provision of RMC services was implementation of SBM-R^©^quality improvement approach; facilities that implemented the approach showed higher level of RMC compared to those who did not. SBM-R^©^ was one of the quality improvement approaches designed to promote RMC reviewed by Bowser and Hill in the 2010 landscape analysis exploring evidence for mistreatment of women in facility based childbirth [39]. Integrating RMC in quality improvement approaches is important in order to improve care for women. Experience of care is an integral part of the WHO’s Quality of Care Framework for Maternal and Newborn Health [40] and RMC improves the experience of care.

### Mistreatment of women

Article IV of the UN’s universal rights of childbearing women document states that every woman has the right to be treated with dignity and respect [41]. In this study, more than a third of the women observed in delivery were not treated with respect, that is, they experienced at least one form of D&A, defined as physical abuse, verbal abuse, violation of privacy and abandonment. In observational studies, physical abuse (slapping/hitting) is expected to be low because of a potential observer effect. In this observational study however, the level of D&A was high compared to an exit interview of women conducted in four sub-counties and Nairobi, Kenya, which reported that 20% of women experienced any form of D&A [42]. However, it was low compared to the prevalence of D&A found in a study using exit interviews conducted in four health facilities in Addis Ababa, Ethiopia, in which 98% of women reported at least one form of D&A [43, 44]. Given the similar cultural contexts, we believe that there might have been some observational effect reducing the prevalence from what it might have been had there been no observers, though one cannot rule out an actual effect of the intervention without further research designed to rule out observer effects.

*Physical abuse* (woman being slapped or hit) was reported in 9% of the observations. This is much higher than observations of care in Tanzania where 2.7% of women living with HIV and 4.7% of women who were not HIV positive were physically abused in labor [45]. Levels of observed physical abuse in this study were also higher than those reported by four client exit interview studies in sub-Saharan Africa [43, 46]. The reason for high rates of physical abuse even in the presence of an external observer was unexpected and needs further investigation as to why health workers are committing such actions. Part of the reason could be rationalization of physical abuse by health providers, with the belief to ensure safety of newborn. In a qualitative study conducted among midwifery students in Ghana and health workers in Nigeria, some students and health workers mentioned it was necessary to hit women to gain compliance [47, 48].

In this study, eight percent of women were *verbally abused* by health providers. This was a little higher than an observational study in a hospital in Tanzania, where providers used non-dignified language with 5.6% and shouted at 6.6% of HIV-negative women while taking their medical history [45]. An exit interview study conducted in Ethiopia and Kenya showed 14% women in Addis Ababa hospitals [43] and 18% of women in Kenya were verbally abused [42]. Reasons for health providers verbally abusing laboring women were not explored in this study but qualitative study in Tanzania suggested coming too early or too late for delivery, wearing old dirty dresses and not pushing strongly were some of the reasons why women were verbally abused by providers [32]. A study in Ghana with midwifery students revealed that both students and their preceptors do not know how to encourage women to push or to open their legs [48].

The rate of verbal abuse observed was less than in client exit interview reports [42] [43]. Much work is needed to eliminate verbal abuse by health providers; treating every woman with respect and dignity is a human right issue.

Though there were factors found to be related to positive treatment of women in labor, assessment of socio-demographic and institutional related factors on the observed mistreatment of women showed that none of the hypothesized factors were significantly associated. This may be related to a greater emphasis on promoting positive behaviors in the quality interventions than on eliminating negative ones, though this requires some investigation. Because we generally think of positive and negative treatment of women as being inversely related to each other and doing one would negate the other, it seems that this was not necessarily the case. Some additional analysis of the relationship between RMC practices and mistreatment of women behaviors may provide useful insight to clinicians, trainers and policy makers.

### Strengths and limitations

A strength of this study is that it is one of the few that has explored prevalence of mistreatment of women through observation. Most studies conducted on mistreatment of women used client exit interviews to measure mistreatment of women, which may underestimate prevalence due to recall bias. The data collectors who observed provider-client interaction observation were clinicians experienced in BEmONC services, or independent consultants who worked in universities or other health facilities outside their permanent work stations.

Another strength of this study was that it covered both hospitals and health centers in the four major regions of Ethiopia, which strengthens its ecological validity. The study also has a number of limitations. Its main limitation is the cross-sectional design, which precludes any conclusion of causal effect. We found associations between some provider and facility-related factors and RMC but cannot conclude that these factors caused RMC. Another study limitation was the possible Hawthorne effect, in which providers will show acceptable behavior during service provision because they know that they are being observed. This effect usually diminishes with each observation and each provider was observed more than once. Also, we can not ignore the potential measurement error caused by differences in understanding among observers. To minimize the potential measurment error, highly experienced assessors who were national trainers of BEmONC training, who received 5 days of training for the observer role and were actively supervised. Lastly, the observation tool used in this study was not validated in Ethiopia as was the tool recently developed in Ethiopia [49]. However, the study team discussed each item in the tool with participants in the data collectors training. It was useful for the observation guides to collect information on both positive and negative behaviors.

## Conclusion

MNH program managers and health professionals’ educational institutions should consider the role of gender and profession on the practice of RMC services. More studies are needed to understand the individual, community, health provider and health facility related factors that affect experience of mistreatment of women in Ethiopia. Preservice education for the maternal health workforce (covers all cadre that work in maternity unit) needs to have RMC as a core area that deserves emphasis. Health care providers were uncomfortable allowing women to deliver other than lying down at their backs. MOH should condider strengthening the training in alternative birthing positions as part of inservice training and preservice education. In addition, inservice training as well as preservice education programs for health workers need to incorporate counselling and communication skills with women in labor. Making delivery beds available that allow alternative birth position in health facilites need to be prioritized. The study team also recommends MOH to consider the role of quality improvement approaches that incorporate providers’s behavior on compassionate and respectful care needs to be implemented across facilites in Ethiopia. Moreover, MOH should establish or strengthen the exiting systems that foster accountability to the public and forms of redress when providers do not meet standards. Finally, the study team recommend health institutions should create greater awareness with the public on the levels of RMC that they should create systems to handle and address complaints.

## Abbreviations

BEmONC: Basic Emergency Obstetrics and Newborn Care
CI: Confidence interval
D&A: Disrespect and abuse
MNH: Maternal and Newborn Health
MOH: Ministry of Health
OR: Odds ratio
RMC: Respectful Maternity Care
SBA: Skilled Birth Attendant
SBM-R^©^: Standards Based Management and Recognition
